# Effects of group sports activities on physical activity and social interaction abilities of children with autism spectrum disorders

**DOI:** 10.3389/fpsyg.2024.1496660

**Published:** 2025-02-06

**Authors:** Yu Xing, Shuaibin Huang, Yatong Zhao, Xueping Wu

**Affiliations:** ^1^School of Physical Education, Hainan University, Haikou, China; ^2^Hainan Provincial Key Laboratory of Sports and Health Promotion, Key Laboratory of Emergency and Trauma, Ministry of Education, The First Affiliated Hospital of Hainan Medical University, Hainan Medical University, Haikou, China; ^3^School of Physical Education and Training, Shanghai University of Sport, Shanghai, China

**Keywords:** autism spectrum disorder, children, group sports activities, physical activity, social interaction ability

## Abstract

**Introduction:**

Group sports activities have been demonstrated to have an impact on the physical activity and social interaction abilities of children with autism spectrum disorder (ASD).

**Methods:**

Thus, this work, taking different types of group sports as the primary variable, explored the impact of 12-week group sports activities on the physical activity and social interaction abilities of children with ASD. A quasi-experimental design was used to divide 21 children with ASD into Experimental group (*N* = 11) and Control group 1 (*N* = 10), while healthy children of the same age were selected as Control group 2 (*N* = 12). The experimental group performed group sports activities for 60 min/time, 4 times/week, for a total of 12 weeks, while the control group maintained the traditional sports activities of Peizhi School. Physical activity was monitored using a three-axis accelerometer (Model: ActiGraph GT3X+), and social interaction ability was measured using the playground observation of peer engagement (POPE) observation scale to evaluate the social interaction states of children in the experimental group after the physical activities.

**Results and discussion:**

After the intervention, the sitting time of children in the experimental group was significantly reduced (*t* = −12.735, *p* < 0.001, Cohen *d* = 2.75), and the time of moderate and high-intensity physical activity was significantly increased (*t* = −8.79, *p* < 0.001, Cohen *d* = 1.82). In social interaction ability, the duration of loneliness was significantly reduced (*t* = −2.567, *p* < 0.017, Cohen *d* = 0.57), and the duration of joint participation (*t* = −3.009, *p* < 0.007, Cohen *d* = 0.02) and the regular game (*t* = −2.511, *p* < 0.026, Cohen *d* = 0.46) were significantly increased, respectively. 4 weeks after the intervention, the sedentary behavior and loneliness of the experimental group both continued to decrease. Group physical activities can improve the physical activity levels and social interaction skills of children with ASD and have a good effect on the maintenance.

## Introduction

Autism spectrum disorder (ASD) is a developmental disorder characterized by neuropsychological and behavioral defects, including autism, Asperger’s syndrome, and childhood disintegrative disorder. A typical presentation of the ASD symptom profile based on the DSM-V criteria, emphasizing two core characteristics: (1) persistent deficits in social communication and social interaction across multiple contexts, including difficulties in social reciprocity, nonverbal communicative behaviors, and developing, maintaining, or understanding relationships; and (2) restricted, repetitive patterns of behavior, interests, or activities, such as stereotyped movements, inflexible adherence to routines, highly restricted interests, or hyper/hyporeactivity to sensory input (Shultz et al., 2018; Kuo et al., 2022). Especially, ASD is often accompanied by a high prevalence of motor impairments, affecting approximately 70% of individuals diagnosed. Additionally, ASD often occurs before the age of 3, and the irreversible abnormal development of the brain leads to the inability to live independently in adulthood. According to a report by the Centers for Disease Control and Prevention (CDC) in 2023, one in 8-year-old children (2.76%) was diagnosed with autism (Hirota and King, 2023), while a study in the Chinese Journal of Reproductive Health in 2022 showed that the prevalence of ASD in children aged 0 to 6 in my country was 1.8% (Zhao et al., 2023). Due to the unknown cause of the disease, there is no specific clinical drug. With the surge in the number of children with autism and the increasing incidence rate year by year, their physical and mental health problems have attracted widespread attention from all walks of life at home and abroad. Finding effective intervention methods to improve autism is an important issue that needs to be solved urgently at home and abroad.

Sports are widely used in the rehabilitation of physical and mental diseases of children and adolescents due to their low cost, easy integration into the education system, and good effects. These motor deficits not only limit participation in physical activities but also exacerbate difficulties in navigating social environments, where physical engagement often plays a pivotal role. Furthermore, children with ASD are more likely to exhibit sedentary behaviors, which increase the risk of associated comorbidities such as obesity and metabolic disorders. They have also become one of the preferred non-drug interventions for ASD. Studies have shown that physical activity participation is an important factor affecting children’s physical health. Low physical activity levels in children with autism will also promote their sedentary behavior habits, which in turn aggravates their social isolation and shows the risk of social interaction and communication interaction disorders (Nguyen et al., 2021). However, these problems do not disappear with age. In recent years, more and more studies have begun to examine the impact of sports and health on the social interaction ability of children with autism. For school-age children with ASD, schools are the main venue for children’s physical activities. Group sports activities based on children’s motor development characteristics create an environment for ASD children to participate in physical activities and increase interaction and communication with peers. Physical activity levels have a positive impact on social interaction ability. At the same time, physiological mechanisms and learning/developmental mechanisms believe that sports intervention can not only change the functional connectivity of the brain’s motor neural network, but also change the activities of social and cognitive areas (Wang et al., 2021). In this context, promoting physical activity emerges as a critical intervention strategy, addressing not only the reduction of sedentary lifestyles but also mitigating the long-term health risks linked to these conditions. By focusing on physical activity, particularly in structured and socially interactive settings like group sports, we may uncover pathways to enhance both physical well-being and social integration for children with ASD.

Currently, group sports activities are widely used in children and adolescent health intervention. Zu Zeyuan implemented group intervention in the form of games and found that scientifically designed group games can cultivate children’s abilities to pay attention, imitate, interact, cooperate and compete, allowing them to learn and develop in a relaxed and pleasant environment (Zu et al., 2022) The results of a meta-analysis show that physical activities can significantly improve the social communication skills, motor skills, and loneliness of children and adolescents with ASD (Chen et al., 2023). Howells et al. used football intervention in a community environment and found that the football skills of children with ASD improved significantly, and the improvement of football skills of ASD children with poor social skills was more obvious (Howells et al., 2021). Qin Luxin and others conducted physical activity intervention on an ASD child and found that the child’s physical activity level and social interaction ability were improved after using peer intervention method (Qin et al., 2023). However, due to the limitations of children’s physical and cognitive abilities in many educational schools in China, the effectiveness of group sports activities is uneven. Therefore, more research is necessary to clarify how group sports activities affect children with ASD. Based on previous research, this study designed group sports activities suitable for children with ASD based on fundamental movement skills, combined with simple and interesting sports games and yoga exercises, aiming to explore the impact of group sports activities on the physical activity and social interaction ability of children with ASD, and further to provide new methods and theoretical basis for clinical sports rehabilitation intervention for children with ASD.

## Participates

This study selected a special education school in Shanghai and a special education school in Wuhu City, Anhui Province as sample recruitment sites. After completing the double screening of the test, the recruitment criteria are as follows: (1) aged 7–10 years old, after child type (holding a medically certified autism diagnosis certificate, based on DSM-V criteria and confirmed through standardized assessments conducted by licensed medical professionals or clinical psychologists), and meeting the CARS diagnostic criteria; (2) no physical, visual and auditory disabilities; (3) no medication or other motor intervention during the intervention; (4) the guardian knew the content of the intervention before the intervention and signed the informed consent. The exclusion criteria are as follows: (1) other diseases that restrict physical activity (such as heart disease, hypertension, and asthma); (2) complex neurological diseases (such as Angelman syndrome and phenylketonuria); (3) the number of absences is less than 90%. Notedly, the detailed methods for both the recruitment and exclusion criteria were detailed in the Supporting Information. Based on these criteria, 24 (7 girls and 17 boys) children with ASD were selected. Among them, 3 children withdrew due to taking too long leave (less than 90%) or failing to complete the test. Finally, 21 children with autism (16 boys and 5 girls) were selected and randomly divided into the experimental group (*N* = 11) and the control group 1 (*N* = 10). At the same time, healthy children of the same age were selected as the control group 2 (*N* = 12). In addition, in order to ensure the personal privacy of each child, all children were uniformly numbered. The test content and process of this study have passed the human experiment ethics review of the Ethics Committee of Shanghai Institute of Physical Education, and the registration number is 102772020RT054.

### Intervention implementation

#### Intervention content and arrangements

In order to ensure that the implementation of the intervention program does not affect the normal teaching order of the special education school (such as Chinese/morality and law/mathematics/painting and handicraft) and considering the similarity of daily teaching, the implementation of the intervention program was finally scheduled for September 2022 to January 2023. The characteristics of the exercise prescription refer to the American College of Sports Medicine. The duration of children’s exercise is 20–30 min/time or 45–60 min/time, 3–5 times/week, the intensity is 50–75% of the maximum heart rate, and the exercise heart rate is maintained within the range of 50–75% of the maximum heart rate (maximum heart rate = 220-actual age) (Geslak, 2017). At the same time, referring to Bahrami, Liu Yinghai and others for the program time, as the number of exercises of autistic children increases, the enthusiasm for exercise participation gradually increases, and the exercise time is adjusted (Bahrami et al., 2012; Liu et al., 2006). Finally, the exercise prescription of the experimental group and the control group was set at 60 min/time, 4 times/week, 4 times/week, for a total of 12 weeks. The details are shown in Tables 1, 2. The experimental group of this study adopted medium-to-high intensity intervention, setting the maximum heart rate per minute to 64% ~ 95%, and used the Bohaotong Polar scale (Beijing Bohaotong Technology Development Co., Ltd.) for full monitoring (Chen et al., 2023; Wang and Yang, 2023). The children did not participate in similar extracurricular courses.

**Table 1:** Time and content distribution of basic motor skills program for the experimental group.

No.	Duration	Content	Form	Target
1	4 min	Classroom routine	Group	Awareness of rules, social interaction
2	10 min	Warm-up activities	Group	Warm-up, attention, social interaction
3	10 min	Skills review (Line up, walk, run, jump, catch, throw and bat)	Team	Attention, social interaction
4	15 min	Social Story skills (Stories about motor skills)	Group/team/individual	Attention, classroom environment adaptation
5	15 min	Skill games (Line up, walk, run, jump, catch, throw, bat)	Group/team/individual	Basic motor skills learning, social interaction
6	6 min	Relaxation exercises (yoga/meditation) and review of teaching	Group	Attention, social interaction

**Table 2:** Specific implementation content of the fundamental movement skills course for the experimental group.

Itemx	Subject
Formation	① Fixed-point standing position + upper limb movement; ② Horizontal team changes + upper and lower limb movement; ③ Vertical team + waiting task in turn
Walk and run	① Complete the specified distance walking or jogging on the marking line; ② High-leg running; ③ Relay running; ④ Circle running; ⑤ “Z” line running;
Step	① Cross the marking line; ② Continuously cross the marking line; ③ Cross the small hurdles with one foot alternately; ④ Cross the small hurdles with one foot alternately and continuously
Jump	① Simple opening and closing jumps; ② Alternate standing on one foot; ③ Alternate high jumps with one foot; ④ Fixed-point jump relay; ⑤ Alternate running and jumping
Catch the ball	① Catch the ball thrown by yourself or others; ② Catch the ball that bounces 1–2 times after landing; ③ Catch the ball thrown by yourself or others Different distances of balls; ④ Alternate catch and throw
Bounce the ball	① Two-handed pat; ② Two-handed continuous pat; ③ One-handed alternating pat; ④ One-handed alternating continuous pat
Throw the ball	① Two-handed chest throw; ② One-handed hand throw (distance, position); ③ One-handed hand throw against the wall
Roll the ball	① Two-handed chest throw; ② One-handed ground roll (distance, position); ③ One-handed wall roll
Kick the ball	① Kick the ball barehanded in place; ② Kick the ball in place; ③ Run-up kick; ④ Run-up kick (distance, position)
Hit the ball	① One-handed barehanded single-handed hit in place; ② One-handed lead-up hit + follow-through; ③ Two-handed barehanded hit in place; ④ Two-handed lead-up hit + follow-through

#### Intervention guidance

##### Social story method

American psychologist Carol Gray designed it based on teaching children to understand social situations (Liang and Yu, 2022). This study is based on the social story method, in which teachers write short stories and specifically tell the time, place and person of the event. The theme of the story is selected based on the intervention goal, describing appropriate behaviors and attitudes that may appear in different social situations (such as “obeying the rules,” “saying hello,” “learning to share”), and how others respond to these social behaviors.

##### Critical response teaching method

Proposed by Koegel et al. (1987) based on the learning characteristics of autistic children, it is a natural teaching strategy based on Applied Behavior Analysis (ABA) (Kim et al., 2019). In behavioral intervention for autistic children, classroom critical response teaching (CPRT) is rated as one of the most scientifically proven methods. This method has been widely used in language, behavioral and social training of autistic children, and has achieved remarkable results (Lei and Ventola, 2017). In this study, before the intervention, professional training researchers conducted theoretical training on “Classroom Critical Response Teaching” for the teachers who implemented the intervention. Before the specific implementation of each CPRT strategy, simulated teaching practice was conducted in a teacher-student 1:1 format, as shown in Table 3. The researcher scored the fidelity of the CPRT strategy, and the teacher’s implementation fidelity was evaluated during the intervention process, with weekly feedback and adjustments to ensure the quality of the intervention.

**Table 3:** Teaching strategy - overhand throw teaching example.

Teaching strategies	Curriculum guidance	Implementation strategies
Children’s attention	When children arrive at the designated location, teachers use stories or situations that are of interest to children or close to daily life, and embed the learned skills.	Establish children’s awareness of rules and improve their attention. By standing at a fixed point, tell stories close to daily life, and show pictures or videos to attract children’s attention.
Clear and appropriate instructions	Teachers and children use task cards, with clear instructions, simple and easy to understand, and intonation.	The teacher demonstrates, prompts the key words of the action, repeats the key words during the practice, and the instructions are clear.
Combination of easy and difficult tasks	Keep the skill learning part and the improvement part at 50%.	Provide tasks of different difficulty levels according to children’s abilities and actual performance, and adjust the difficulty level from bare hands to object control, distance, and time.
Shared control	Teachers provide children with opportunities to choose independently.	Children choose the materials they like and choose which task to start with.
Multiple cues	Teachers use different pictures, videos or stories to prompt teaching points.	Post pictures in the children’s practice area for observation and learning.
Immediate reinforcement of outcome strategies	Provide immediate feedback based on the actual performance of children.	If children respond correctly, encourage them to continue practicing.
Dependent on outcome	Motivate children to strengthen with positive feedback.	If children respond correctly, lead them to participate in their favorite games.
Reinforcement of attempts	Provide meaningful feedback immediately based on children’s behavior.	If children follow instructions and complete the task, reward them in time to reinforce their attempts.

#### Intervention quality control

15 teachers of the school took turns to serve as the main teacher and assistant teacher to be responsible for the specific implementation of the program. Each intervention was implemented by 4 teachers (2 main teachers and 2 assistant teachers). Each teacher has many years of experience in teaching severely autistic children which refers to Level 3 severity based on the diagnostic criteria outlined in the DSM-V, indicating a need for very substantial support in social communication and restrictive, repetitive behaviors. The entire program implementation process was recorded. Two researchers observed the teaching execution of the main teacher and assistant teacher in class and recorded the children’s activities. After each observation, a summary observation report was made and filed, and the teaching implementation status was promptly fed back to the main teacher and assistant teacher. In order to ensure the quality of the intervention, the teachers were guided by the Shanghai Institute of Physical Education Adaptive Sports Team and the Shanghai Baoshan District Autism Rehabilitation Center.

### Measurement

#### Symptom assessment

The Childhood Autism Rating Scales (CARS) was developed by Schopler et al. (1980). The scale has 15 assessment items and is accompanied by detailed evaluation rules (Pang et al., 2018). The scale is scored according to a four-level standard. The scale has a total of 60 points, and the scoring standards for each level are “age-appropriate behavior” (total score less than 30 points), “mild abnormality,” “moderate abnormality” (total score 30–36 points, and less than 5 items are less than 3 points) and “severe abnormality” (total score greater than or equal to 36 points, and at least 5 items are greater than 3 points).

#### Physical activity level

ActiGraph GT3X+ was used to assess the daily sedentary, low-intensity, moderate-intensity, and high-intensity physical activity time of children with autism. The measurement method was as follows: before the test, the researcher initialized the instrument and set the test time, then distributed the accelerometer to each subject, demonstrated how to wear it and explained the relevant precautions. Each subject was required to wear the accelerometer on the right hip for 1 week, and informed the guardian in the informed consent form that the accelerometer should be worn from waking up in the morning until the child fell asleep, except during bathing or swimming. After wearing, the data were collected uniformly. The researchers used ActiLife (Version 5.5.5) to export the original data, and screened the data according to the standard that each data included at least 5 school days and 2 weekend wearing days and the wearing time was not less than 8 h per day. Finally, Kinesoft software was used for analysis, and physical activity data were extracted and processed. After data selection, the relevant analysis mainly selected medium and high intensity physical activities.

#### Social interaction abilities

This study used the playground observation of peer engagement (POPE) scale to assess changes in the subjects’ social interaction skills. The POPE scale is an interval behavior coding system used to examine children’s interactions and social behaviors with peers on the playground. The POPE scale includes 7 social interaction states: loneliness (the participant is alone and has no peers within 1 meter), proximity (the target child plays alone within 1 meter and does not participate in activities), watching (the participant is aware of another peer 1 meter away, but does not participate in activities), parallel (the participant and peer are parallel), parallel perception (the participant and peer participate in the same activities and are aware of each other), joint participation (the participant and peer participate in social behaviors with each other), and games with rules (participating in game activities with other children according to specified game rules). This tool has been successfully used in various peer social observation studies on school playgrounds, sports rooms, and playgrounds. Independent observers continuously observed children with autism on the playground or in the exercise room for at least 15 min, recording every 1 min. The first 40 s were used to observe the state of social interaction, and the last 20 s were used to code and record the qualitative description of the students’ social behavior. Then, the time spent on the seven social interaction states within 15 min and the total observation time were statistically analyzed in percentage. The consistency and reliability of the observations of the two observers were tested by Kappa statistics, and the average was taken. Two raters randomly and independently coded 20% of the observation data, maintaining an average reliability of 0.89 (0.83–0.96) between raters. Two trained researchers used the POPE scale to conduct a 4-week follow-up assessment of the social behavior of children with autism in the first, sixth and twelfth weeks of the exercise intervention. The quantitative and qualitative data generated were used to describe the trend of changes in the social behavior of children with autism during the intervention.

### Statistical analysis

Statistical analysis was performed using SPSS 23.0. The measurement data conforms to the normal distribution and satisfies the homogeneity of variances, expressed as (xˉ ± s). One-way analysis of variance is used for comparison between groups, pairwise comparison is performed using LSD test, and paired sample *t* test is used for comparison within the group. Use Cohen’s *d* to represent the effect size of the *t* test: 0.2 ~ 0.5 is a small effect, 0.5 ~ 0.8 is a medium effect, and *d* > 0.8 is a large effect, where significance level *α* = 0.05.

### Physical movement

**Figure 1:**
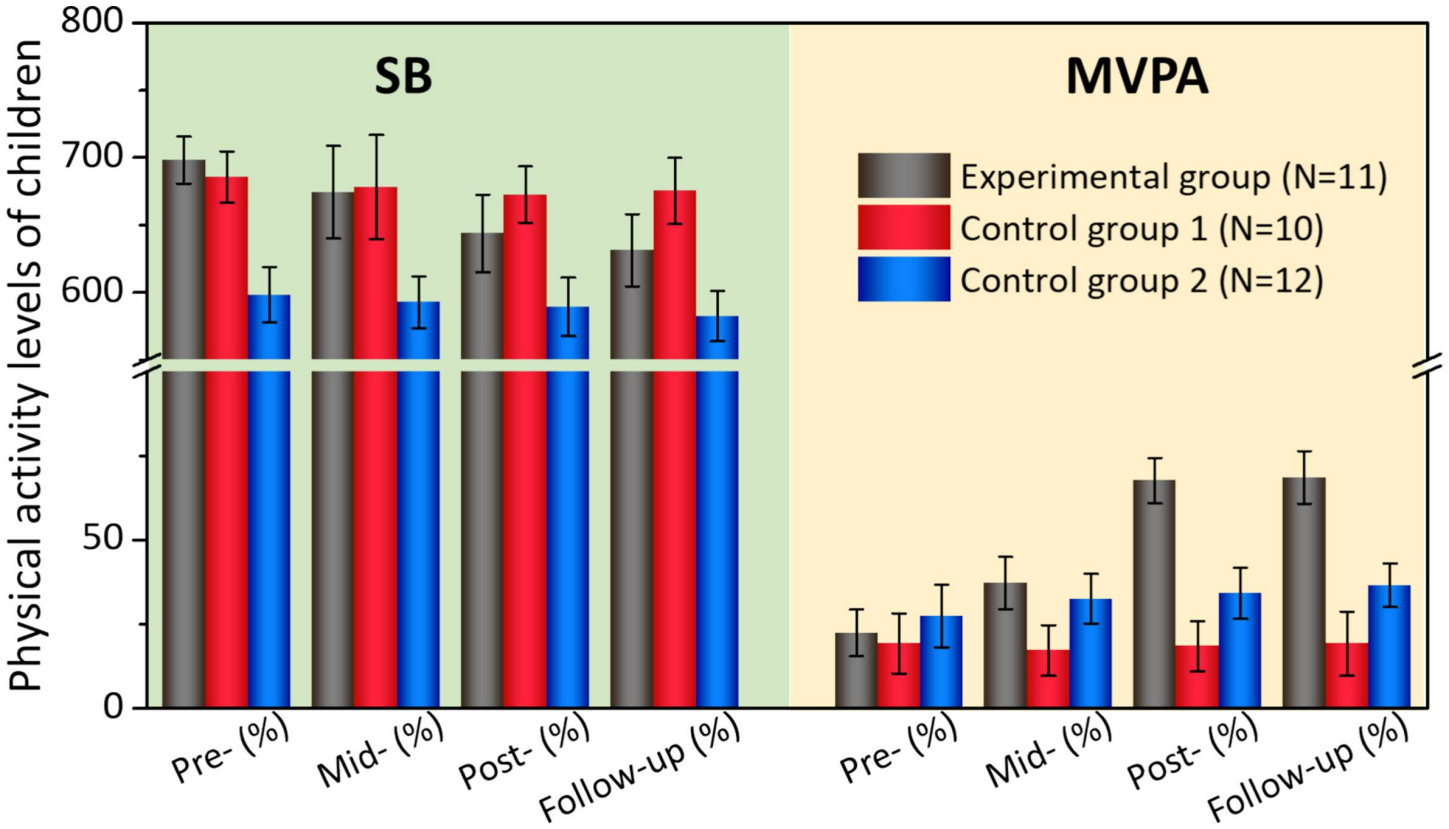
Physical activity levels of children before and after intervention.

Figure 1 and Table 4 compare the physical activity levels of children before and after intervention. It can be found that there was no significant difference in physical activity levels between the experimental group and the control group (*p* > 0.05) before intervention. The sitting behavior of control group 2 was lower than that of experimental group and control group 1 (*p* < 0.01), and the level of moderate and high-intensity physical activity was higher than that of experimental group and control group 1 (*p* < 0.05). After the intervention, the sedentary behavior in the pre-test and post-test, mid-test and post-test of the control group 1 was higher than that of the experimental group and control group 2 (*p* < 0.01), and the level of medium- and high-intensity physical activity was lower than that of the experimental group and control group 1 (*p* < 0.05); the pre-test and post-test sedentary behavior of the experimental group (*t* = −12.735, *p* < 0.001, Cohen *d* = 2.75) was lower than before the intervention, and the moderate and high-intensity physical activity (*t* = −8.79, *p* < 0.001, Cohen *d* = 1.82) was higher than before intervention, the pretest and posttest sitting behavior of Control 1 (*t* = −2.874, *p* = 0.041, Cohen *d* = 0.47) and Control 2 (*t* = −2.513, *p* = 0.027, Cohen *d* = 0.64) were lower than before the intervention. 4 weeks after the intervention, the sitting behavior of the experimental group (*t* = −3.56, *p* = 0.034, Cohen *d* = 0.57) continued to decrease (Table 4).

**Table 4:** Comparison of children’s physical activity levels before and after intervention.

	SB				MVPA			
	Pre-	Mid-	Post-	Follow-up	Pre-	Mid-	Post-	Follow-up
F	34.672	15.027	18.585	17.172	2.875	2.172	3.585	6.438
P	<0.001	0.045	0.037	0.039	< 0.001	0.043	0.031	0.02

### Social interaction abilities

**Figure 2:**
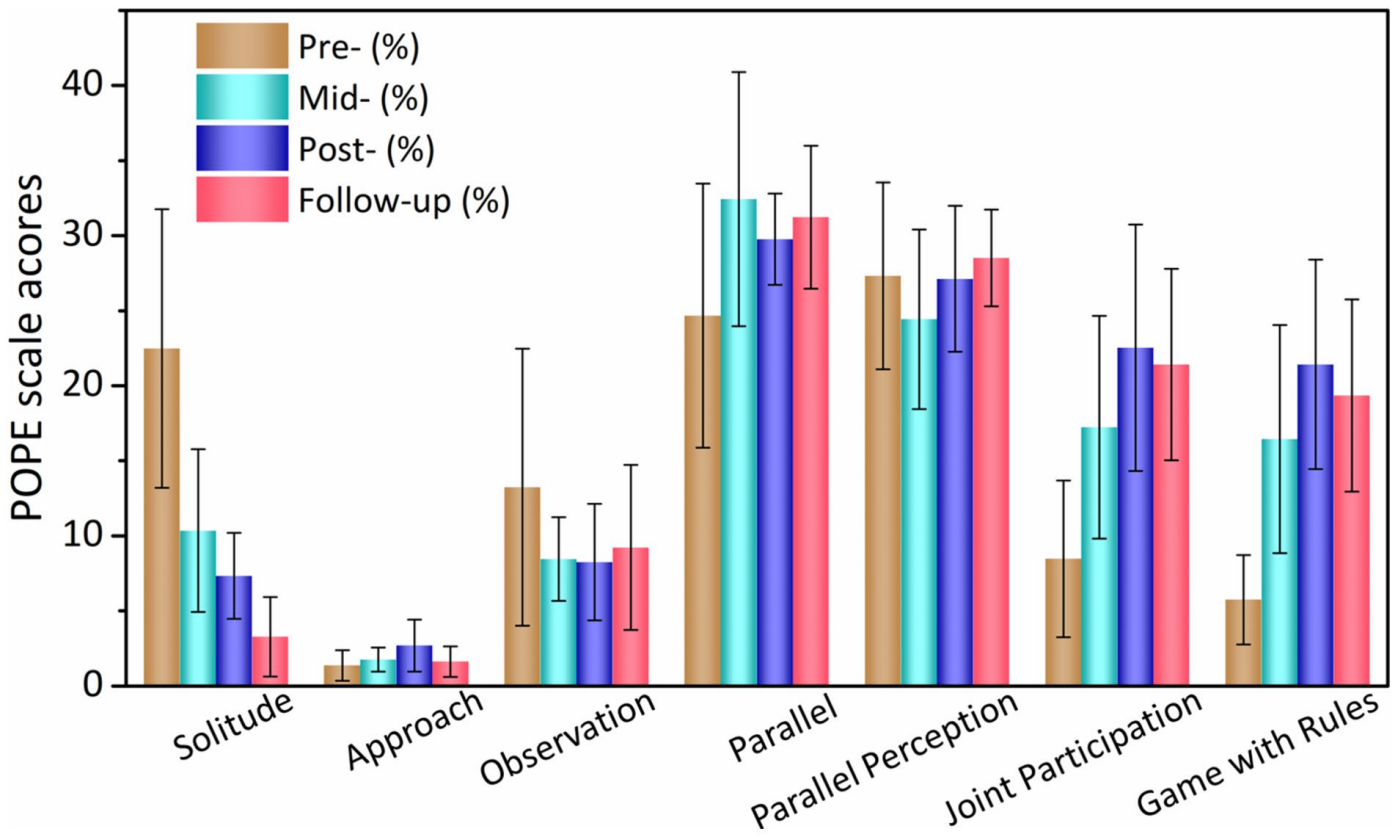
Children’s POPE scale scores in the experimental group.

The POPE scale, as shown in Figure 2 and Table 5 was used to observe and record the social communication abilities of children with ASD in physical activities during the intervention period. After the intervention, the length of lonely state in physical activities of ASD children in the experimental group was significantly reduced in pre-test and post-test (*t* = −2.567, *p* = 0.017, Cohen *d* = 0.57), and the duration of the joint participation (*t* = −3.009, *p* = 0.037, Cohen *d* = 0.02) and the regular game state (*t* = −2.511, *p* = 0.026, Cohen *d* = 0.46) were significantly reduced, respectively. 4 weeks after the intervention, the duration of autistic state during physical activities in the experimental group continued to decrease (*t* = −2.57, *p* = 0.018, Cohen *d* = 0.37) (Table 5).

**Table 5:** Changes in POPE scale scores of children in the experimental group during the intervention period.

	Solitude	Approach	Observation	Parallel	Parallel perception	Joint participation	Game with rules
F	7.43	0.93	2.17	1.72	0.714	3.72	0.91
P	0.017*	0.725	0.452	0.427	0.516	0.037*	0.026*

* *p* < 0.05.

## Discussion

This study explored the impact of 12 weeks of group physical activity on physical activity levels and social interaction skills in children with ASD. The results found that 12 weeks of group sports activities significantly improved the physical activity levels (sitting and moderate-to-high-intensity physical activity levels) and social interaction abilities of autistic children. Dong and Ketcheson formed a structured exercise program by improving basic motor skills (Dong et al., 2019; Ketcheson et al., 2016). The final research results verified that exercise intervention based on basic motor skills can promote the motor skills, physical activities and social functions of children with ASD development, which is consistent with the results of this study.

Children with ASD are more likely to be overweight, obese, and physically inactive than their peers without disabilities (Curtin et al., 2010). Patients with ASD usually do not participate in sports activities, resulting in continued decline in participation in sports activities as adults (Garcia-Pastor et al., 2019). Therefore, children and adolescents with ASD are more likely to be overweight and less physically active than children and adolescents without disabilities (McCoy and Morgan, 2020). The group sports activities designed in this study are aimed at children’s movement development, combined with the characteristics of group activities, focusing on children’s physical activity participation and social interaction abilities. In each sports activity, the direction, route, speed, distance, strength and Changes in venue equipment allow children to learn fundamental movement skills, arouse children’s interest in “playing,” make children “move,” and provide the best environment for participation and practice in sports activities.

A lack of interest in social interaction is an inherent symptom of ASD and one of the most common symptoms involves reacting appropriately and responding accordingly to others. Children with ASD generally display characteristic behaviors, such as preferring to play alone, avoiding contact with others, reacting inappropriately to others, avoiding eye contact with others, lack of facial reactions, poor understanding of social cues, and inability to establish peers appropriately. Relationships, a lack of social–emotional reciprocity and shared enjoyment, and difficulties accepting other people’s perspectives and maintaining conversations with others. Judging from the results of this study, the duration of loneliness in children with ASD during sports activities was significantly reduced, and the duration of joint participation and rule-based games were significant. This shows to a certain extent that group sports activities can improve the social communication abilities of children with ASD. This is basically consistent with the research results of Dong and Bremer (Dong et al., 2021; Bremer et al., 2015). Through meta-analysis, scholars found that group sports activities have a significant positive impact on the social interaction ability, communication ability, motor skills and autism severity of autistic children (Huang et al., 2020). After conducting physical activity intervention on 50 autistic children, it was found that the social skills and social interaction of autistic children improved overall in the middle and late stages of training, and there were also significant improvements in communication, cooperation, social interaction and self-control (Zhao and Chen, 2018). Research in the field of neurochemistry shows that brain-derived neurotrophic factor (BDNF) plays a role in the acquisition of learning skills in autistic individuals (Chang and Kochel, 2020). The increase in BDNF levels after exercise intervention promotes neuroplasticity in ASD individuals, thereby improving the social interaction and cognitive level of ASD individuals. The brain organization hypothesis holds that physical activities require the joint participation of the visual, auditory, and proprioceptive cortices, which are all related to the function of the cerebellum. Therefore, it can be assumed that during exercise, the cerebellum of ASD individuals is fully stimulated and developed, promoting sports participation and ultimately improving their social interaction ability.

In team sports activities, the main teacher provides social stories and critical response teaching methods, introducing and encouraging children’s social behaviors (such as eye contact, nodding, clapping, speech) and orderly activities through social stories. At the same time, taking CPRT as the teaching guiding principle, CPRT focuses on the “core area,” which can include many development areas, such as communication skills, joint attention, etc. Once the intervention produces changes, secondary development areas will also have a chain effect. This intervention not only helps sports participation, but also has a positive impact on communication and social areas. During the intervention process, the 1:1 teacher-student ratio also enables timely feedback on the teaching effect. Exercise intervention is characterized by multi-sensory engagement, thus contributing to the occurrence of high levels of social motivation and socially engaged behaviors, especially in the areas of social skills, communication, timely response and frequency of expression. Movahedi et al. found that karate training significantly improved social dysfunction in children with ASD. It is worth noting that this effect can last for at least 1 month (Movahedi et al., 2013). In a follow-up study, Bahrami et al. from the same research team mentioned above examined the impact of karate training on communication deficits in children with ASD. The study also found that compared with the control group, the communication skills of children in the experimental group were significantly improved, and the effect. It can last for at least 1 month, while subjects in the control group did not experience the above effects (Bahrami et al., 2016). Overall, existing research supports that exercise can improve the social skills of individuals with ASD, and some intervention effects can last at least 1 month. At the same time, the medium-to-high-intensity group sports activities used in this study may further stimulate nerves and improve the intervention effect.

### Research limitations and prospects

This study has some limitations, mainly including the following aspects: (1) Due to the large individual differences among children with ASD, the sample size of this study is small, which to some extent affects the statistical analysis effect, and the conclusions drawn have certain limitations. Later studies can conduct regular group sports activities on the basis of expanding the sample size. (2) After the intervention, there is a lack of generalization effect testing for children with ASD. Later studies can use “functional linkage-advanced development” as a practice paradigm to improve the acceptance of the intervention in this group, while paying attention to tracking the sustained and generalized effects of the intervention, strengthening school-family-community linkage, creating opportunities and environments for children with ASD to participate in sports, and constantly exploring sports patterns suitable for children with ASD.

## Conclusion

Participation in group sports activities has a significant effect on reducing sedentary behavior, increasing the time of medium to high intensity physical activity, and improving social interaction skills in children with ASD. Compared with traditional sports activities in special schools, by improving basic motor skills, structured sports programs have a better effect on improving their physical activity levels and social interaction skills. In the future, special schools can provide targeted, operational, and regular adaptive sports activities for children of different categories, strengthen follow-up investigations on the effects of exercise intervention on individuals with autism, and ultimately achieve the goal of establishing guidelines for exercise intervention for individuals with ASD.

## Data Availability

The raw data supporting the conclusions of this article will be made available by the authors, without undue reservation.
