# Supplementary material for: Effects of group sports activities on physical activity and social interaction abilities of children with autism spectrum disorders
Source: Front Psychol. 2025 Feb 6;15:1496660. doi: 10.3389/fpsyg.2024.1496660 (PMC11840914; doi:10.3389/fpsyg.2024.1496660)
Supplement: Supplementary file 1 [file Data_Sheet_1.docx]

Supporting Information

**Effects of Group Sports Activities on Physical Activity and Social Interaction Abilities of Children with Autism Spectrum Disorders**

Yu Xing^1, 2,^ ^[[1]](#footnote-1)^*, Yatong Zhao^1^, and Xueping Wu^3,^ ^[[2]](#footnote-2)^*

*1 School of Physical Education, Hainan University, Haikou 570228, China*

*2 Hainan Provincial Key Laboratory of Sports and Health Promotion, Key Laboratory of Emergency and Trauma, Ministry of Education, The First Affiliated Hospital of Hainan Medical University, Hainan Medical University, Haikou 571199, China*

*3 School of Physical Education and Training, Shanghai University of Sport, Shanghai 200438, China*

## **Participates**

**(1) The recruitment criteria are as follows:**

1) aged 7-10 years old, after child type (holding a medically certified autism diagnosis certificate), and meeting the CARS diagnostic criteria.

First, it provided a detailed explanation of how the autism diagnosis was medically certified. The diagnosis was based on DSM-V criteria and confirmed through standardized assessments conducted by licensed medical professionals or clinical psychologists. Tools such as the Childhood Autism Rating Scale (CARS) and comprehensive psychological evaluations, including the Wechsler Intelligence Scale for Children, were utilized. Medical records and prior evaluations were also reviewed to ensure accurate classification. These details clarify the criteria and process for diagnosis and certification.

Second, we elaborated on the use of the CARS in the methods section to provide greater clarity. The CARS is a standardized tool that evaluates 15 areas of behavior, including social relationships, verbal and nonverbal communication, sensory responses, and adaptive behaviors. Each item is scored on a 4-point scale ranging from "normal for age" to "severely abnormal," with scores aggregated to provide an overall severity rating. A total score of 30 or above indicates a diagnosis of autism, with higher scores reflecting greater symptom severity. This tool was used during screening to ensure consistent and reliable assessment of participants.

Third, the statistical analysis section has been updated to detail how assessment items and scores from the diagnosis and CARS were utilized. Specifically, CARS scores, including subscale ratings for key behavioral domains (e.g., social interaction, communication, sensory responses), were treated as independent variables to assess their predictive value in relation to the dependent variables of physical activity levels and social interaction abilities. These data points were integrated into a multiple regression analysis to explore the relationships between the severity of autism symptoms and the outcomes of the group sports interventions.

2) no physical, visual and auditory disabilities;

This recruitment criteria specified that participants should not have any physical, visual, or auditory disabilities to ensure the feasibility and focus of the study interventions. This exclusion criterion was applied to eliminate potential confounding factors that might interfere with participants' ability to engage in group sports activities or accurately measure the effects of these activities on physical activity levels and social interaction abilities. For example, physical disabilities might limit participation in certain motor tasks, while visual or auditory impairments could affect communication or sensory integration within the group sports context. This approach aimed to maintain a consistent baseline across participants and ensure that the study specifically addresses the impact of group sports on children with autism spectrum disorder.

3) no medication or other motor intervention during the intervention;

The recruitment criteria specified that participants should not be undergoing any medication or other motor intervention during the study period. This requirement was established to eliminate potential confounding variables that could influence the study’s outcomes. Medications, particularly those targeting neurological or behavioral conditions, may alter physical activity levels or social interaction abilities, while motor interventions such as physical therapy or specialized training programs could independently impact motor skills and related behaviors. By ensuring that participants were not receiving these treatments concurrently with the group sports intervention, we aimed to attribute observed changes in physical activity and social interaction specifically to the intervention itself.

4) the guardian knew the content of the intervention before the intervention and signed the informed consent.

The recruitment criteria required that the guardians of all participants fully understood the content, purpose, and procedures of the intervention prior to its commencement and provided their informed consent. Before enrollment, guardians were given detailed information about the study, including the types of group sports activities involved, the expected duration and frequency of sessions, and the specific objectives of improving physical activity levels and social interaction abilities in children with autism. This ensured transparency and allowed guardians to make an informed decision about their child’s participation. By signing the informed consent form, guardians acknowledged their understanding of the study and agreed to their child’s involvement, in compliance with ethical research standards.

**(2) The exclusion criteria are as follows:**

1) other diseases that restrict physical activity (such as heart disease, hypertension, and asthma);

The exclusion criteria specified that participants should not have any other diseases that could restrict physical activity, such as heart disease, hypertension, or asthma. This criterion was established to ensure the safety of all participants and to prevent health conditions that might limit their ability to engage in the group sports activities included in the intervention. These conditions could also serve as confounding factors, making it difficult to isolate the effects of the sports intervention on physical activity levels and social interaction abilities. By excluding individuals with such conditions, the study aimed to maintain a controlled environment where the outcomes could be attributed specifically to the intervention itself, while also prioritizing the health and well-being of the participants.

2) complex neurological diseases (such as Angelman syndrome and phenylketonuria);

The exclusion criteria specified that participants should not have complex neurological diseases, such as Angelman syndrome or phenylketonuria. These conditions were excluded because they often involve unique developmental, behavioral, or physical challenges that could significantly influence the outcomes of the intervention and confound the study’s findings. For example, Angelman syndrome is characterized by severe intellectual disability, motor impairments, and unique behavioral profiles, while phenylketonuria, if untreated, can lead to cognitive deficits and other neurological complications. Including participants with such conditions could introduce variability unrelated to autism spectrum disorder (ASD) or the group sports intervention, making it challenging to isolate the intervention’s specific effects on physical activity and social interaction abilities. This exclusion ensured a more homogenous sample, focusing on children with ASD as the primary population of interest.

3) the number of absences is less than 90%.

The exclusion criteria specified that participants must attend at least 90% of the scheduled intervention sessions to remain in the study. This criterion was established to ensure the consistency and reliability of the intervention’s effects on the study outcomes. A high attendance rate was necessary to provide sufficient exposure to the group sports activities, allowing for meaningful evaluation of their impact on physical activity levels and social interaction abilities. Participants with excessive absences may not have received the full benefits of the intervention, potentially leading to inconsistent data or skewed results. By setting a strict attendance threshold, the study aimed to maintain a controlled and standardized environment for all participants, ensuring the validity of the findings.

1. * Corresponding author.

   *E-mail address*: xingyu@hainanu.edu.cn (Y.X.). [↑](#footnote-ref-1)
2. *E-mail address*: 1030523763@qq.com (X. Wu). [↑](#footnote-ref-2)
